# A High-Performance and Versatile Fluorometer for Chlorophyll a Monitoring

**DOI:** 10.3390/bios15120787

**Published:** 2025-12-01

**Authors:** Tingkai Zhang, Yee Lyn Sim, Jianchao Luo, Shuming Ye

**Affiliations:** Key Lab of Biomedical Engineering, Ministry of Education, College of Biomedical Engineering and Instrumentation Science, Zhejiang University, Hangzhou 310027, China; 12215013@zju.edu.cn (T.Z.);

**Keywords:** chlorophyll a, fluorescein sodium, modulation, fluorescence

## Abstract

Chlorophyll a (Chl a) monitoring is vital for aquatic ecosystem assessment but is challenged by low signals and interference. Addressing this, we introduced balanced maximum entropy m-sequence modulation for Chl a fluorescence detection, developing a novel high-sensitivity sensor offered in configurations for both portable and deep-sea applications. The sensor achieved outstanding limits of detection (LOD) of 4.17 ng/L (fluorescein sodium, FS) and 4.82 ng/L (spinach-extracted Chl a), significantly surpassing commercial instruments. It also features a wide dynamic range (0–500 μg/L FS, R^2^ = 0.9983), excellent long-term stability with negligible drift, measured system response T90 < 24 s, and low power consumption (40 mA working/0.5 mA standby). Critically, a near 1:1 fluorescence response gain between FS and extracted Chl a under the sensor’s specific configuration was experimentally demonstrated, validating FS as a high-precision proxy standard and simplifying future calibration. Field experiments in West Lake confirmed the sensor’s ability to accurately track spatial variations consistent with the standard spectrophotometric method, achieving highly consistent quantitative results (within 3% relative error) at half sampling locations. In summary, the sensor developed in this study, due to its outstanding performance, promises to provide a reliable and efficient new tool for high-precision portable and deep-sea in situ chl a monitoring.

## 1. Introduction

As a key pigment required for photosynthesis in algae and phytoplankton, chlorophyll concentration can intuitively reflect the abundance of these aquatic plants [1]. Chlorophyll a (Chl a), the primary pigment among chlorophylls involved in photosynthesis, is itself a fluorescent compound and serves as a biomarker for chlorophyll concentration in water [2]. Chl a fluorescence monitoring is not only an important means for measuring phytoplankton biomass and photophysiological status but also a vital approach to understanding the deep chlorophyll maximum. Furthermore, it is key to understanding and quantifying the role of deep chlorophyll in carbon budget processes [3]. Consequently, Chl a has become a core indicator in marine and lacustrine research, climate change studies, and aquatic ecosystem management. Accurate fluorescence monitoring of Chl a is of great significance for in-depth research into major scientific issues such as the global carbon cycle and marine primary productivity [4,5].

Laboratory methods such as high-performance liquid chromatography and spectrophotometry are commonly used for detecting chlorophyll concentration in water, with spectrophotometry being recognized as the standard method. However, these laboratory techniques require sampling, involve complex and time-consuming procedures, and fail to meet the demand for real-time monitoring [6,7,8,9]. While common remote sensing approaches offer high temporal resolution, they are limited to monitoring chlorophyll on the sea surface and cannot detect potentially active phytoplankton communities in deeper waters [3]. Fluorescence spectroscopy, due to its advantages of high sensitivity, rapid response, and non-invasiveness, has emerged as one of the mainstream in situ monitoring methods for chlorophyll in water. Portable monitoring devices based on fluorescence spectroscopy enable in situ chlorophyll monitoring with excellent spatial and temporal resolution, facilitating efficient data collection and analysis [10]. Nevertheless, the chlorophyll extraction process is complex, and Chl a is unstable under prolonged light exposure, readily degrading into byproducts like chlorophyll b and pheophytin, which affects fluorescence detection [11]. To optimize the calibration process for chlorophyll fluorescence sensors, fluorescent dyes such as fluorescein sodium, Rhodamine WT Red, and Rhodamine B have been used as calibration substances [11,12,13]. Among these, fluorescein sodium is widely utilized for calibrating chlorophyll fluorescence sensors due to its emission spectrum being most similar to that of phytoplankton cultures and its high stability [13,14,15].

The fluorescence signal of Chl a is extremely weak compared to background noise such as sunlight. To achieve high-precision detection, researchers have widely adopted modulation-demodulation techniques to enhance the signal-to-noise ratio (SNR) of fluorescence detection [16,17,18,19]. Currently, modulation methods employed in in situ fluorescence sensors primarily fall into two categories: single-frequency modulation and pseudo-random sequence modulation based on maximum-length sequences (m-sequence) [18,19,20,21]. The first, single-frequency modulation, is simple but suffers from a critical vulnerability: if an interference signal’s frequency coincides with the modulation frequency, the detection system can collapse. The second category, pseudo-random sequence modulation (e.g., m-sequence), offers a broader spectrum, but its energy is still primarily concentrated in the DC and low-frequency bands. This creates significant overlap with common, strong interference sources such as sunlight and power-line frequencies, leading to a low SNR after filtering. While advanced techniques like frequency shifted spread spectrum (FSSM) have been developed to move the m-sequence spectrum to higher frequencies, the resulting spectrum is often not uniform [22]. This non-uniformity means that a high probability of system collapse still exists if a specific interference frequency overlaps with one of its concentrated spectral peaks [23]. Therefore, the complex environments encountered during field detection and in situ measurements of Chl a fluorescence demand sensors that maintain maximum robustness while achieving high sensitivity and high-quality concentration detection.

To address the aforementioned challenges, we introduced the balanced maximum entropy m-sequence (BMEm) into Chl a fluorescence detection for the first time and designed a novel portable chlorophyll sensor based on the BMEm sequence [23]. This sensor achieved high stability while demonstrating a chlorophyll limit of detection (LOD) of 4.82 ng/L, which is half that of the current best-performing commercial sensors. In this work, fluorescein sodium was used as the standard substance for sensor calibration, achieving an LOD of 4.1 ng/L and establishing the concentration correspondence between fluorescein sodium and extracted chlorophyll. Additionally, the sensor features a dynamic detection range of 0–500 μg/L for fluorescein sodium and rapid response characteristics. This sensor is capable of both portable detection and mobile observation in deep-sea environments up to 6000 m. When used in conjunction with the Chlorophyll Monitor software, it allows for convenient data visualization and instrument function management. We conducted field detection and sampling in West Lake, verifying the consistency between the sensor’s results and those obtained by spectrophotometry. Therefore, this study promises to provide more precise information support for future portable detection and deep-sea in situ chlorophyll monitoring, contributing to research on the global carbon cycle, marine primary productivity, and other significant areas.

## 2. Materials and Methods

### 2.1. Modulation Sequence of Chlorophyll Sensor

**Figure 1:**
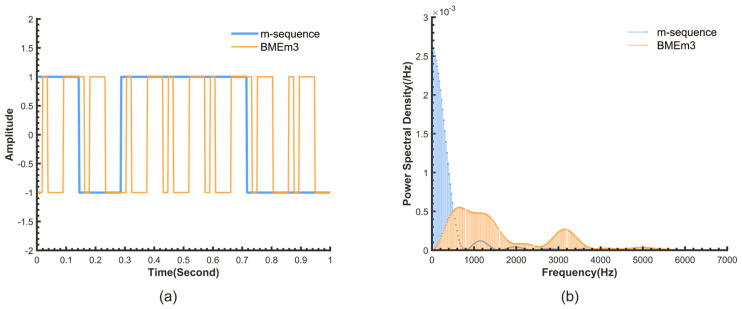
(**a**) Time domain waveforms of the BMEm3 and the corresponding m-sequence (N = 7 bits, Tc = 1/7 s). (**b**) Power spectral density of the m-sequence and BMEm3 (N = 31 bits, Tc = 1/800 s).

In pseudo-random sequence modulation, the m-sequence, which is a pseudo-random binary sequence generated by a linear-feedback shift register, is widely used in detection fields due to its excellent autocorrelation and spectral characteristics. Based on these properties, researchers have further modified and optimized it, enabling the modulated sequence to achieve stronger anti-interference performance while retaining the excellent characteristics of the m-sequence. As a next-generation sequence designed based on the m-sequence, BMEm can further enhance the robustness of the chlorophyll sensor without increasing computational complexity and power consumption. This sequence is generated using our proposed ‘maximum entropy construction method’. This method performs an exclusive OR operation between an m-sequence and a de Bruijn sequence, which serves as the maximum entropy sequence. The de Bruijn sequence is characterized by its high information entropy. For binary sequences of the same length, higher information entropy implies a greater number of possible combinations in the time domain, meaning that the sequence contains all possible combinations of a given length within its subsequences. This in turn leads to more random frequency components in the frequency domain. The complete algorithm for this construction method is detailed in our prior work [23]. The comparative time-frequency domain characteristics of the resulting BMEm sequence and the original m-sequence are shown in Figure 1. According to the definition of the BMEm sequence, in this paper, an 8-bit length maximum entropy sequence was used to modulate the m-sequence. We named the resulting BMEm sequence BMEm3.

As can be seen from Figure 1, the m-sequences with lengths N = 7 and 31 bits and chip widths Tc = 1/7 s and 1/800 s, respectively, are segmented into a more randomBMEm3 by the maximum entropy sequence (Figure 1a). Its spectrum is shifted to higher frequencies, and the energy distribution of the spectrum becomes more uniform (Figure 1b), thereby reducing the risk of interference signals coinciding with the modulation signal, which could lead to system collapse, thus enhancing the robustness of the detection system.

### 2.2. Mechanical and Optical Design of the Sensor

To meet different detection needs, we designed two configurations for the chlorophyll fluorescence sensor, primarily differing in their power supply method: a built-in battery configuration and an external battery configuration, both based on the same optical and circuit design. The built-in battery configuration, having an extended body, is relatively less portable and suitable for deployment on platforms such as autonomous underwater vehicles and human-occupied vehicles for deep-sea in situ chlorophyll observation. The external battery configuration is suitable for portable chlorophyll detection on the surface water like lakes and rivers, as well as for laboratory detection. These two sensor configurations have no difference in detection performance or optical structure. Regarding the optical structure, the sensor employs two high-precision LEDs with a peak wavelength of 470 ± 5 nm as excitation sources. The optical path utilizes classic orthogonal geometry, where the excitation and receiving paths are perpendicular to each other.

**Figure 2:**
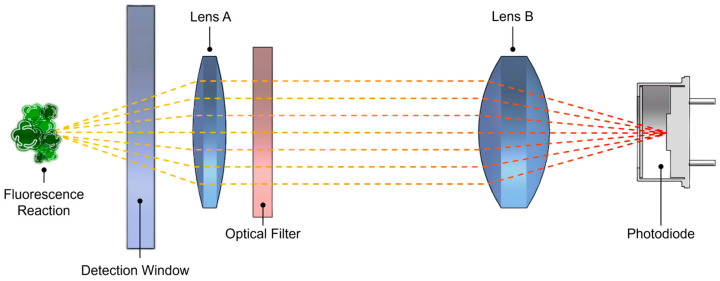
Schematic diagram of the dual-lens conjugate focusing design.

The receiving path features a dual-lens conjugate focusing design (Figure 2), effectively reducing stray light entering the detection area of the photodiode (S1336, Hamamatsu Photonics, Hamamatsu, Japan). Concurrently, to achieve effective chlorophyll detection, a filter with a center wavelength of 685 nm and a full width at half maximum of 30 nm is placed between the dual lenses. At the bottom of the sensor, a circular PEEK material is used as a baffle to minimize external light interference, further enhancing the sensor’s reliability in practical applications. To improve the sensor’s pressure resistance and reliability, the housing is machined from titanium alloy material. Standard cyclic pressure tests have verified that the sensor can operate normally in environments up to 70 MPa, meeting the application requirements for sea trials at depths of 6000 m.

### 2.3. Sensor Detection System

**Figure 3:**
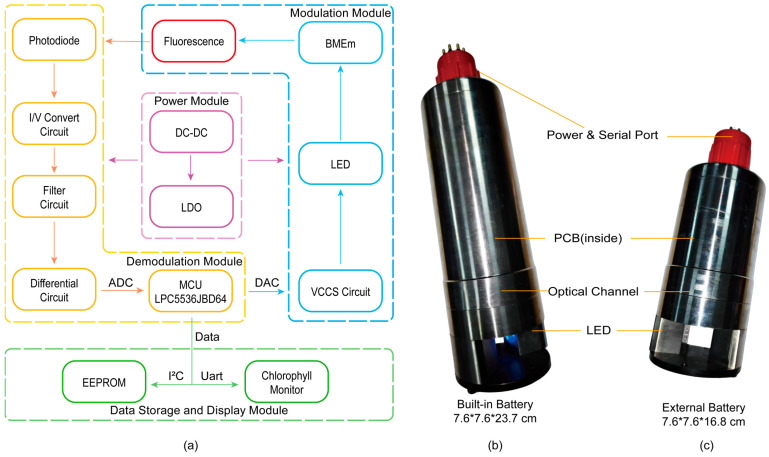
(**a**) The block diagram of the chlorophyll sensor. (**b**) The built-in battery configuration of chlorophyll sensor. (**c**) The external battery configuration of chlorophyll sensor.

The sensor’s detection system, as shown in Figure 3a, mainly comprises four modules: the power module, modulation module, demodulation module, and data storage and display module.

**Figure 4:**
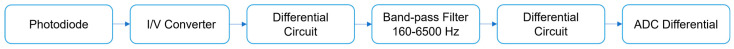
Block diagram of the analog signal conditioning chain.

The sensor can be powered by a 12–24 V source. The external power supply undergoes step-down processing via DC-DC converters and Low-Dropout regulators (LDOs) to supply power to the analog and digital circuits of the sensor. The modulation module is primarily implemented through the microcontroller unit (MCU) chip LPC5536, controlling a constant current source circuit. By adjusting the digital-to-analog converter (DAC) output voltage, a constant current drive for the LEDs on both sides is achieved, and the excitation light modulation output is controlled by turning the LEDs on and off, ultimately obtaining the corresponding modulated fluorescence signal. After being focused by lenses and filtered, the fluorescence signal, primarily within the 670–700 nm wavelength range, enters the photodiode and is converted into an electrical signal. Through the I/V conversion stage implemented by a transimpedance amplifier, the weak current signal generated by the photodiode is converted into a voltage signal. Following this first stage and low-pass filtering, the signal undergoes differential amplification. The amplified signal passes through a conditioning circuit to filter out interference signals outside the 160–6500 Hz range. Subsequently, the signal is sent to an operational amplifier preceding the analog-to-digital converter (ADC) for secondary differentiation, further eliminating common-mode interferences such as ground noise. Finally, the MCU’s 16-bit successive approximation register ADC samples the output of the secondary differentiation at a speed of 320 kHz (Figure 4). In subsequent software calculations, these data are converted to an equivalent 25-bit resolution [20]. This high resolution is achieved computationally by adopting an oversampling and averaging strategy, similar to the design philosophy of Delta-Sigma ADCs. Specifically, the high-speed raw samples undergo sequential digital processing, including correlation demodulation and extensive accumulation. This process allows the final output data to attain a numerical precision equivalent to that of a 25-bit ADC. Subsequently, digital cross-correlation is performed between the sampled data and the locally stored BMEm sequence. This demodulation process coherently accumulates the desired synchronous fluorescence signal while averaging out asynchronous noise to achieve a high SNR. Finally, digital signal processing is performed to derive the numerical value representing the current Chl a concentration. After data processing, these final data are transferred via I2C to an EEPROM for data backup, fulfilling the requirements for self-contained detection. When the sensor is connected to a computer, these data can be transmitted via RS-485 to our custom-developed Chlorophyll Monitor software (V1). The Chlorophyll Monitor integrates essential functions such as serial communication, data storage, data display and replay, real-time clock synchronization, and instrument mode selection. In practical use, the software supports different data acquisition strategies depending on the operating mode: in online mode, it directly displays and records real-time fluorescence data. In self-contained mode, it functions as a data retrieval tool, reading historical data and time stamps from the EEPROM for post-analysis. Additionally, the software facilitates switching between the sensor’s working modes, including self-contained, online, and standby modes.

The sensor can be powered by a 14.8 V, 3000 mAh lithium battery(Figure 3b,c). When the supply voltage is 14.8V, the operating current in both self-contained and online modes is 40 mA, allowing for approximately 75 h of operation. As the built-in battery configuration is not easily detachable, the sensor can be switched to standby mode when not in operation. In this mode, the standby current is 0.5 mA, which is 1/800th of the normal working current, enabling a standby time of up to 250 days and significantly reducing the need for charging.

### 2.4. Calibration of Chlorophyll Sensor

The linear calibration experiments for the Chl a sensor were conducted under normal laboratory lighting conditions without any shielding. In the linear calibration experiment, ultrapure water was used as the solvent, with an initial solution volume of 800 mL. During the experiment, corresponding amounts of fluorescein sodium solution were added to prepare fluorescein sodium solutions with final concentrations of 0, 1, 2, 5, 10, 20, 50, 100, 200, and 500 μg/L. This concentration range was selected to characterize the sensor’s full linear dynamic range. After the solutions were thoroughly mixed, the instrument’s output values were recorded (n = 50). This sample size was chosen to ensure a statistically stable mean for linear regression and to simultaneously quantify measurement precision.

### 2.5. LOD Experiment of Chlorophyll Sensor

To comprehensively evaluate the detection performance of this sensor, LOD experiments were conducted using both standard fluorescein sodium solution and spinach-extracted chl a solution as analytes. All experiments were performed in an ultrapure water solvent environment under normal laboratory lighting conditions, without additional light shielding measures. This unshielded setup was intentionally chosen to verify the sensor’s robustness against ambient light interference, demonstrating the effectiveness of the BMEm modulation algorithm in suppressing background noise. In the fluorescein sodium LOD experiment, ultrapure water was taken as the initial solvent, with an initial volume of 800 mL. During the experiment, a 0.4 mg/L fluorescein sodium stock solution was quantitatively added to prepare a series of fluorescein sodium solutions with final concentrations of 0, 10, 20, 40, and 60 ng/L. After the solutions were uniformly mixed, the instrument output values were recorded (n = 50).

On the other hand, considering that pure Chl a standards are expensive and chemically unstable, this study employed spinach leaf extract as a readily preparable and cost-effective benchmark substance to evaluate the sensor’s detection limit. Spinach leaves with petioles removed were placed in a grinding device, and 90% acetone solution was added for grinding and extraction. The extract was then transferred to centrifuge tubes and allowed to extract in the dark at 4 °C for at least 2 h. Subsequently, the sample was centrifuged at 4000 r/min for 10 min. After filtration, the supernatant was taken, and its absorbance at specific wavelengths was measured using a spectrophotometer. The Chl a concentration of the extract was then calculated according to the equations for 90% acetone solvent by Jeffrey & Whitney [24]. Finally, it was diluted to obtain a 0.2 mg/L Chl a extract solution as the test solution for the LOD experiment. In this experiment, ultrapure water was used as the initial solvent with an initial volume of 800 mL. During the experiment, the 0.2 mg/L Chl a extract solution was quantitatively added to prepare a series of Chl a solutions with final concentrations of 0, 10, 20, 40, and 60 ng/L. After the solutions were uniformly mixed, the instrument output values were recorded (n = 50).

### 2.6. Stability Experiment

In the stability experiment for this sensor, ultrapure water was used as the test solution. The sensor operated continuously for 24 h, and its output values were recorded during this period. The experiment was conducted under normal laboratory lighting conditions at room temperature, without additional light shielding or temperature control measures. By conducting the test in an open light environment, we aimed to simulate real-world background noise conditions and further validate the interference-rejection capability of the modulation system during long-term continuous operation.

### 2.7. Transient Response Experiment

To accurately quantify the sensor’s transient response in a real-world scenario, a solution step-change experiment was conducted. The sensor was placed in a beaker containing ultrapure water under continuous magnetic stirring to ensure rapid mixing. A quantitative amount of fluorescein sodium stock solution was then instantly added to the beaker to achieve a final concentration of 1 μg/L. The sensor’s real-time output was recorded to determine the time required to reach a stable reading.

### 2.8. Temperature Experiment

To evaluate the sensor’s baseline thermal stability under deep-sea conditions, a dedicated temperature experiment was performed. The sensor was immersed in a beaker containing ultrapure water and focused on instrumental baseline drift. The water temperature was regulated to gradually decrease from 25 °C to 3 °C. This temperature range was selected to simulate the vertical temperature gradient observed during deep-sea deployments in the South China Sea. The sensor’s output was recorded continuously to monitor baseline variations.

### 2.9. Field Application

On 8 April 2025, the prototype was used for chlorophyll detection in the waters of West Lake, Hangzhou, with the detection point located at 30.271° N, 120.131° E. To verify the sensor’s performance, measurements were taken at four locations in West Lake near Wugui Islet, and corresponding water samples were collected. The concentrations of these samples were analyzed on the same day using the spectrophotometric method [10]. During the detection process, the sensor was operated in online mode for portable detection, and the instrument’s detection data were compared and analyzed against the spectrophotometric detection data.

## 3. Results and Discussion

### 3.1. Linear Calibration of Chlorophyll Sensor

To comprehensively evaluate the sensor’s dynamic detection performance, we first conducted a wide-range linear calibration using fluorescein sodium standard solutions, covering a range of 0–500 μg/L.

**Figure 5:**
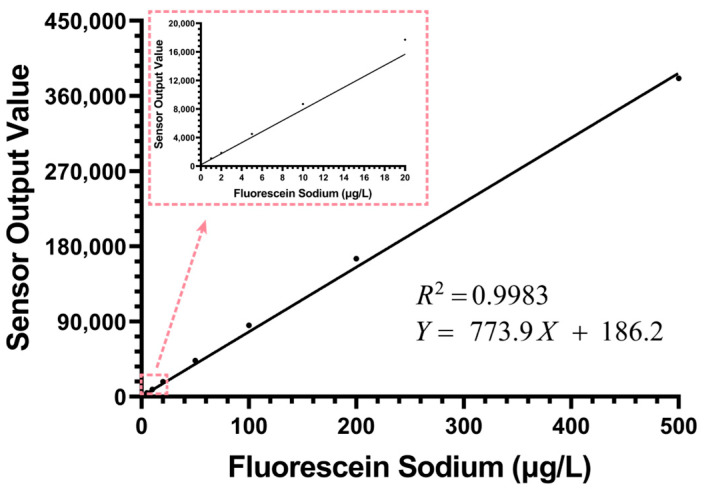
The results of the fluorescein sodium linear calibration experiment for the chlorophyll sensor within the concentration range of 0–500 μg/L (n = 50).

As shown in Figure 5, the sensor exhibited good linear correlation throughout the dynamic range, with a coefficient of determination (R^2^) reaching 0.9983. The fitting equation is *Y* = 773.9*X* + 186.2, where the intercept was constrained to the experimental mean of the blank solution (186.2 counts). The 95% confidence interval for the slope was determined to be 771.5 to 776.3. Additionally, the standard deviation of the blank solution was measured to be 1.72 counts. Based on this high degree of linearity, we could still observe an extremely slight systematic deviation. In the 0–200 μg/L concentration range, the sensor’s actual output values were slightly higher than the fitted line, while at the 500 μg/L high concentration point, its output value was slightly lower than the fitted value. This minor deviation, which only becomes apparent at high concentrations, is a typical manifestation of the inner filter effect (IFE) [25,26]. In applications requiring the pursuit of ultimate precision, non-linear calibration can be used to correct this minor error introduced by IFE, thereby achieving high-quality chlorophyll concentration detection.

### 3.2. LOD Experiment

To comprehensively evaluate the sensor’s capability to detect ultra-low concentration signals, LOD experiments were conducted. To achieve a comprehensive assessment and validation of the instrument’s hardware performance, we used both the highly stable chemical standard, fluorescein sodium, and spinach-extracted Chl a for testing.

**Figure 6:**
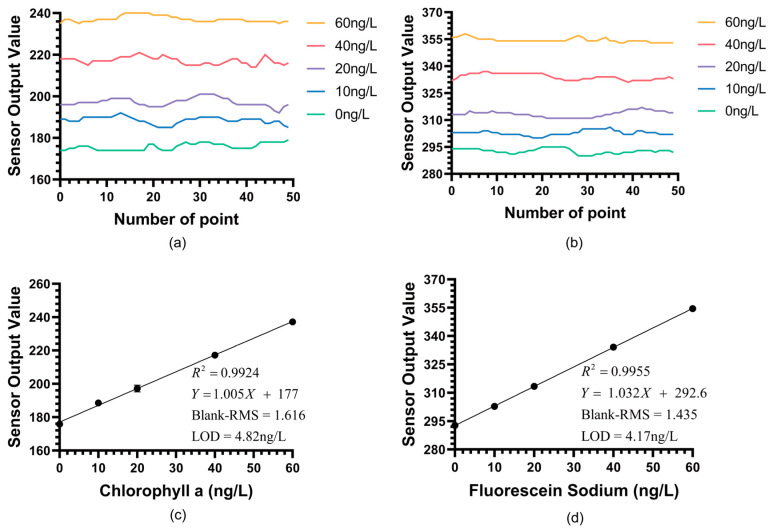
(**a**) Real-time detection results of the Chl a LOD experiment from the instrument output. (**b**) Real-time detection results of the fluorescein sodium LOD experiment from the instrument output. (**c**) The detection results of the Chl a LOD experiment for the chlorophyll sensor within the concentration range of 0–60 ng/L (n = 50). (**d**) The detection results of the fluorescein sodium LOD experiment for the chlorophyll sensor within the concentration range of 0–60 ng/L (n = 50).

Figure 6a,b respectively shows the real-time detection outputs of the sensor for Chl a and fluorescein sodium within the 0–60 ng/L concentration gradient. It is clearly visible from the figures that the sensor’s background noise was extremely low, and it could still produce clear and stable signal responses at concentration steps as low as 10 ng/L. Figure 6c,d presents the linear regression analysis based on these data. The analysis results showed that the low-concentration fitting curve for fluorescein sodium is *Y* = 1.032*X* + 292.6(R^2^ = 0.9953). The standard errors for the slope and intercept were calculated to be 0.0044 and 0.148, respectively. Meanwhile, the fitting curve for Chl a is *Y* = 1.005*X* + 177(R^2^ = 0.9924), with slope and intercept standard errors of 0.0056 and 0.189, respectively.

The extremely low standard errors relative to the coefficient values in these low-concentration fits further confirm the high stability and linearity of the sensor’s performance near the detection limit.

(1) $$\mathrm{LOD}=\frac{sample\;concentration}{S-B}\times RMS\;Noise\times 3$$

Here, S denotes the chlorophyll a sensor output average sample concentration, and B is the average output at blank solution. RMS Noise in this context refers to the sample standard deviation of the instrument’s output signal at blank solution, which accurately represents the noise level for LOD calculation. According to the LOD calculation formula [27], this sensor achieved a detection limit of 4.17 ng/L for fluorescein sodium and 4.82 ng/L for chl a. This result confirms that the BMEm-based chlorophyll sensor possesses an extremely high signal-to-noise ratio and sensitivity.

More importantly, this experiment establishes the quantitative relationship between fluorescein sodium and extracted Chl a. From the fitting curves, it is known that the sensor’s response gain for fluorescein sodium is 1.023, while the response gain for Chl a is 1.005. This near 1:1 gain, while specific to our optical configuration (470 nm excitation, 685 nm reception), is plausibly explained by a compensating spectroscopic trade-off.

Specifically, fluorescein sodium exhibited a high excitation efficiency at 470 nm (near its absorption peak) but low detection efficiency at 685 nm, as the filter only captures the weak tail of its emission spectrum (peak ~515 nm). Conversely, while 470 nm is a standard excitation wavelength for commercial chlorophyll sensors, it is not the peak absorption band for Chl a, resulting in moderate excitation efficiency. However, the detection efficiency is maximized as the 685 nm filter aligns perfectly with the Chl a emission peak. Consequently, these inverse spectral efficiencies offset each other, resulting in a balanced signal output. The primary significance of this finding is the validation of fluorescein sodium as an excellent proxy standard. This is crucial not for replacing essential, water specific field calibration, but for providing a stable, reliable benchmark for instrument manufacturing and hardware inter-comparison, thereby solving the core pain points of expensive and unstable Chl a standards.

### 3.3. Stability Performance

**Figure 7:**
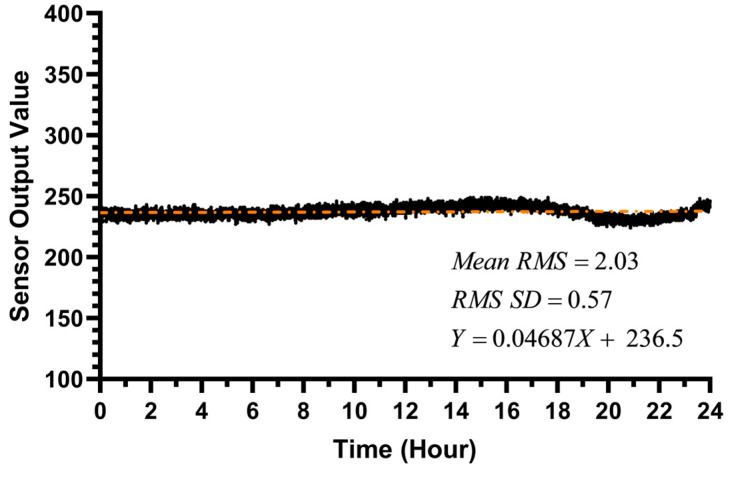
The 24-h stability experiment of the chlorophyll sensor.

To comprehensively assess the sensor’s reliability during long-term continuous operation, a 24-h stability experiment was conducted. As shown in Figure 7, the sensor’s raw output signal remained on an extremely stable horizontal line over 24 h of continuous operation in ultrapure water. To precisely quantify this stability, we performed a linear fit on the drift curve, obtaining a slope of only 0.04687. Based on this fit, the sensor’s theoretical total drift over the entire 24-h period was calculated to be less than 1 count. This value is far below the instrument’s background noise level and is entirely negligible in practical measurements. This directly demonstrates the sensor’s excellent long-term stability, free from significant signal drift caused by component heating or temporal effects.

To further quantify the stability of the sensor’s background noise, an in-depth analysis of the 28,800 data points collected during the experiment was performed. We employed a moving window method, calculating the Root Mean Square (RMS) value for every 50 adjacent data points. Statistical results showed that the average of these RMS values over the entire 24-h period was 2.03 counts, indicating that the sensor’s background noise was suppressed to an extremely low level, consistent with the blank noise (1.635 counts) observed in the LOD experiment. More importantly, the standard deviation of these RMS values themselves was only 0.57. This extremely low standard deviation provides strong evidence that the sensor’s noise level itself is also highly stable and does not fluctuate significantly with time or environmental changes. Therefore, the sensor exhibits excellent long-term stability, which is crucial for field in situ monitoring tasks requiring long-term, self-contained deployment.

### 3.4. Sensor Transient Response

A sensor’s transient response time is a critical metric for evaluating its ability to capture rapid changes in the aquatic environment, such as during vertical profiling.

**Figure 8:**
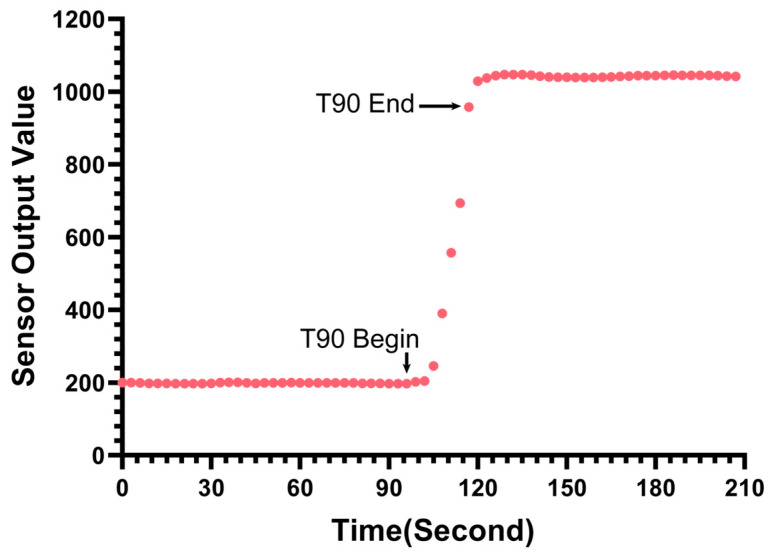
Transient response of the sensor.

Figure 8 illustrates the sensor’s actual response curve during the step-change test from the blank solution to 1 μg/L fluorescein sodium. The start of the addition and the stabilization process are clearly marked. The results indicate that the sensor responds rapidly to the concentration change. The measured T90 response time (the time required for the reading to reach 90% of the final stable value) was approximately 24 s. This confirms that the sensor possesses a fast response capability suitable for profiling applications.

It should be noted that typically, commercial chlorophyll sensors do not explicitly specify the T90 response time in their technical datasheets. Consequently, a direct horizontal comparison with these commercial instruments regarding this specific metric could not be performed in this study.

### 3.5. Effect of Temperature

Thermal stability is a critical performance metric for in situ fluorescence sensors, particularly for applications involving deep-sea profiling where the instrument experiences significant vertical temperature gradients. Variations in ambient temperature can potentially induce electronic drift or optical baseline shifts, compromising data accuracy. To strictly evaluate the sensor’s robustness against such environmental changes, a baseline thermal stability test was conducted.

**Figure 9:**
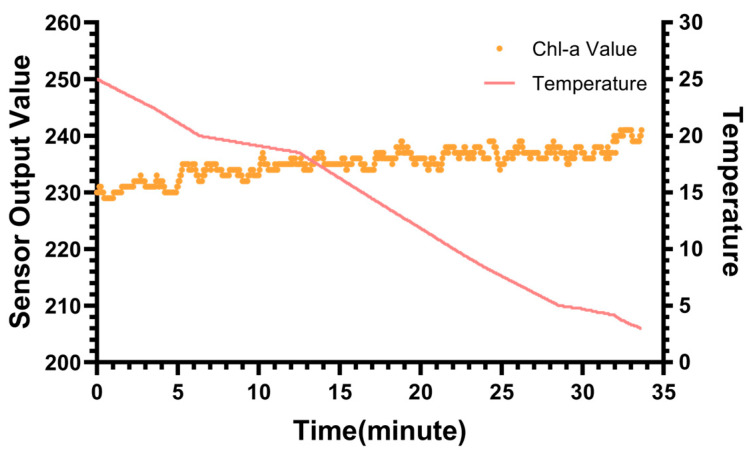
The effect of temperature on the sensor output.

Figure 9 illustrates the results of this test as the temperature decreased from 25 °C to 3 °C, mimicking a deep-sea descent. It can be observed that the sensor’s output remained remarkably stable throughout the cooling process. The maximum fluctuation observed across the entire 22 °C range was limited to approximately 20 counts, corresponding to a chlorophyll equivalent variation of ~20 ng/L. Given the sensor’s LOD (4.82 ng/L), the Limit of Quantitation was approximately 14.5 ng/L. The observed thermal drift was only marginally above this LOQ threshold. This confirms that the temperature-induced baseline shift is comparable to the instrument’s intrinsic quantification limit and is effectively negligible for practical measurements.

### 3.6. Field Experiment in West Lake

**Figure 10:**
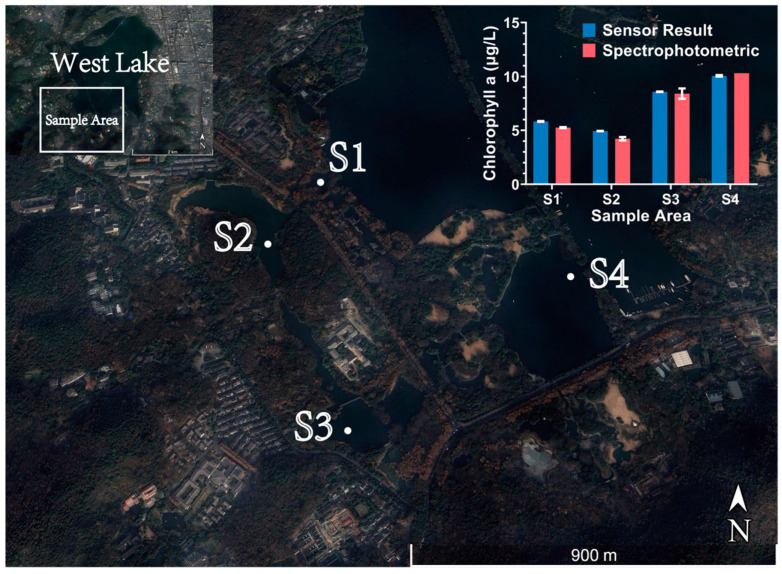
Sampling points (S1–S4) in West Lake and comparison of the sensor results with spectrophotometric measurements.

To verify the practical application performance of this sensor in a real and complex aquatic environment, we conducted in situ detection in West Lake, Hangzhou (sample area shown in Figure 10) and compared the results with the standard laboratory spectrophotometric method using collected water samples from four locations (S1–S4). In the analysis, the Chl a gain determined from laboratory calibration was used to convert the sensor’s readings into detected concentrations, which were then compared with the Chl a concentration values obtained from the spectrophotometric measurements.

The analysis revealed that the sensor’s measurements generally tracked the spatial variations observed by the laboratory method, exhibiting good consistency in relative trends. To ensure statistical reliability, we analyzed the sensor data collected over a stabilized 30 s window at each sampling point. The relative errors for the four locations (n = 10) were calculated as −10.76% (S1), −17.06% (S2), −2.02% (S3), and 2.30% (S4). Concurrently, repeated tests (n = 2) using the spectrophotometric method on water samples from the same locations revealed internal measurement deviations of −0.57% (S1), −2.72% (S2), −4.04% (S3), and 0% (S4), representing a range of 0% to −4.04%.

As visualized by the error bars in the updated Figure 10, the sensor demonstrated superior precision (smaller standard deviation) compared to the spectrophotometric method. While a relatively significant deviation was observed at locations S1 and S2, we attribute this to two primary factors. First, the inherent spatial heterogeneity of the water body likely resulted in the in situ sensor and the sampler capturing slightly different water parcels. Second, and more fundamentally, such discrepancies are inevitable when comparing fluorescence measurements with spectrophotometric results. The detection environment faced by fluorescence detection is far more complex than that of laboratory detection, as it cannot remove interfering substances such as suspended particles and various degradation products through methods like filtration and extraction. Therefore, the signal detected by the sensor is a relatively complex mixed signal, which will naturally exhibit certain differences from the results obtained after pure extraction in the laboratory.

It should be noted that this field experiment in West Lake only analyzed data from four locations and was conducted on a single day (8 April 2025). Therefore, this study serves as a preliminary validation and does not account for seasonal or diurnal variations in chlorophyll fluorescence. If this sensor is to be applied in future scientific research tasks with extremely high precision requirements, more detailed multi-point field calibration specific to the target water may be necessary to establish a more accurate calibration model tailored to the local water characteristics.

Overall, the results of this preliminary field experiment in West Lake are successful. The sensor not only accurately captured the spatial variation trends of Chl a concentration but also achieved quantitative results highly consistent with the standard laboratory method at most locations. This fully validates the sensor’s ability to work stably in real environments and demonstrates its significant potential for practical monitoring applications.

### 3.7. Instrument Comparison

To objectively evaluate our prototype sensor against current commercial instruments, Table 1 provides a detailed comparison of its key performance specifications with four mainstream commercial sensors. Compared to current in situ chlorophyll fluorescence sensors, the most significant advantage of this prototype lies in its LOD. Our prototype achieved a Chl a LOD of 4.82 ng/L. This performance not only surpasses chlorophyll sensors from companies like Turner, Seapoint, and Seabird but is also more than twice as sensitive as the current highest-sensitive commercial chlorophyll sensor, the RBRtridente. This exceptional sensitivity fully demonstrates the significant superiority of our adopted BMEm modulation technique in suppressing background noise and improving the signal-to-noise ratio.

The high sensitivity of the sensor was also cross-validated by the detection results using the fluorescein sodium standard. When the RBRtridente was configured for the standard fluorescein detection channel, its LOD was 10 ng/L, whereas our sensor’s LOD for fluorescein sodium was only 4.17 ng/L. This superiority, demonstrated in a strict fluorescein sodium comparison, strongly corroborates that the 4.82 ng/L chl a detection limit measured using the spinach extract is a true and reliable performance metric.

Furthermore, the sensor also exhibits advantages as a in situ instrument in terms of power consumption and measurement range. The typical operating current of our prototype is 40 mA, which is among the lowest compared to the listed instruments, being only 13% of the Turner sensor’s consumption and better than Seabird’s 50 mA. This is crucial for long-term deployments relying on battery power. Concurrently, the sensor’s 0–500 µg/L detection range also significantly exceeds the ranges of Seabird, Seapoint, and RBR sensors. In summary, while maintaining equivalent pressure resistance and dimensions comparable to commercial sensors, our prototype leads in the three core indicators of sensitivity, power consumption, and range simultaneously.

## 4. Conclusions

This study introduced and validated a novel portable chl a fluorescence sensor employing BMEm modulation. By incorporating the BMEm sequence into chl a fluorescence detection for the first time, combined with low-noise electronic design, the sensor demonstrated significant performance advantages.

Experimental results clearly showed that the sensor possesses extremely high sensitivity. Its LOD for fluorescein sodium and spinach-extracted chl a reached 4.17 ng/L and 4.82 ng/L, respectively, superior to current mainstream commercial sensors. Critically, this research revealed experimentally, for the first time, that under the sensor’s specific configuration, the fluorescence response gains for fluorescein sodium and extracted chl a were nearly 1:1. This finding not only validates the feasibility of using fluorescein sodium as a high-precision proxy standard but also provides an important basis for simplifying future calibration procedures for chlorophyll sensors.

Furthermore, the sensor exhibited excellent performance in other key metrics: it possesses a wide dynamic detection range covering 0 to 500 μg/L fluorescein sodium and demonstrated outstanding long-term stability, with its theoretical baseline drift over 24 h being far below the instrument’s own background noise level, thus negligible in practical measurements. Its measured system response time (T90 < 24 s) and low power consumption (working current 40 mA, standby current 0.5 mA), coupled with two flexible mechanical configurations and a 6000 m depth rating, enable it to fulfill diverse tasks ranging from portable surface water detection to long-term in situ observation in the deep sea. Preliminary field validation in West Lake further confirmed the sensor’s practical application potential. The sensor not only accurately captured the spatial variation trends of chl a concentration, but also achieved quantitative results highly consistent with the standard laboratory method.

In conclusion, the BMEm-modulated chl a sensor developed in this study, by virtue of its high sensitivity, high stability, low power consumption, and precise validation against a proxy standard, contributes a high-performance and extremely promising advanced tool to the field of aquatic environmental monitoring, particularly for deep-sea in situ observation and global carbon cycle research.

## Figures and Tables

**Table 1:** Comparison of chlorophyll sensors.

	Turner USA [28]	Seabird USA [29]	Seapoint USA [30]	RBR Canada [31]	This Prototype
LOD	30 ng/L	20 ng/L	20 ng/L	10 ng/L	4.82 ng/L
Range	0–500 μg/L	0–125 μg/L	0–150 μg/L	0–50 μg/L	0–500 μg/L
Memory (Samples)	60,000	108,000	/	/	32,767
Input Volt (V)	8–30	9–36	8–20	4.5–30	12–24
Current (Typical)	300 mA	50 mA	15 mA,27 mA pk	/	40 mA
Depth (m)	600	600	6000	6000	6000
Dimensions (cm)	10 × 10 × 23	6.3 × 6.3 × 12.7	6.4 × 6.4 × 16.8	6.33 × 6.33 × 5.7	7.6 × 7.6 × 16.8/23.7

## Data Availability

Data are contained within the article.
